# Author Correction: Truncation of mutant huntingtin in knock-in mice demonstrates exon1 huntingtin is a key pathogenic form

**DOI:** 10.1038/s41467-020-19873-9

**Published:** 2020-11-19

**Authors:** Huiming Yang, Su Yang, Liang Jing, Luoxiu Huang, Luxiao Chen, Xianxian Zhao, Weili Yang, Yongcheng Pan, Peng Yin, Zhaohui S Qin, Beisha Tang, Shihua Li, Xiao-Jiang Li

**Affiliations:** 1 Department of Neurology, The First Affiliated Hospital, Sun Yat-sen University, 510080 Guangzhou, China; 2 Guangdong-Hongkong-Macau Institute of CNS Regeneration, Ministry of Education CNS Regeneration Collaborative Joint Laboratory, Jinan University, 510632 Guangzhou, China; 3 Department of Neurology, Xiangya Hospital, Central South University, 410008 Changsha, Hunan China; 4 Department of Human Genetics, Emory University School of Medicine, Atlanta, GA 30322 USA; 5 Department of Emergency, Tongji Hospital, Tongji Medical College, Huazhong University of Science and Technology, 430030 Wuhan, China; 6 Department of Biostatistics and Bioinformatics, Rollins School of Public Health, Emory University, Atlanta, GA 30322 USA; 7 Department of Neurology & Key Laboratory of Hunan Province in Neurodegenerative Disorders, Xiangya Hospital, Central South University, Hunan, China

**Keywords:** Huntington's disease, Molecular neuroscience

Correction to: *Nature Communications* 10.1038/s41467-020-16318-1, published online 22 May 2020.

The original version of this Article omitted from the author list the 11th author Beisha Tang, who is from the ‘Department of Neurology & Key Laboratory of Hunan Province in Neurodegenerative Disorders, Xiangya Hospital, Central South University’. Consequently, the following was added to the Author Contributions: ‘B.T. sponsored H.Y. to do research at Emory University and provided advice on the experiments’. This has been corrected in both the PDF and HTML versions of the Article.

In addition, the original version of this Article contained an error in the author affiliations. Yongcheng Pan was incorrectly associated with Department of Neurology, The First Affiliated Hospital, Sun Yat-sen University, 510080 Guangzhou, China and their affiliation with Department of Neurology & Key Laboratory of Hunan Province in Neurodegenerative Disorders, Xiangya Hospital, Central South University was inadvertently omitted. This has now been corrected in both the PDF and HTML versions of the Article.

