# Supplementary material for: Truncation of mutant huntingtin in knock-in mice demonstrates exon1 huntingtin is a key pathogenic form
Source: Nat Commun. 2020 May 22;11:2582. doi: 10.1038/s41467-020-16318-1 (PMC7244548; doi:10.1038/s41467-020-16318-1)
Supplement: Supplementary file 1 — Supplementary Information [file 41467_2020_16318_MOESM1_ESM.pdf]

Truncation of mutant huntingtin in knock-in mice demonstrates exon1 huntingtin is a key pathogenic form

Yang HM et al

Supplementary Information

Supplementary figures

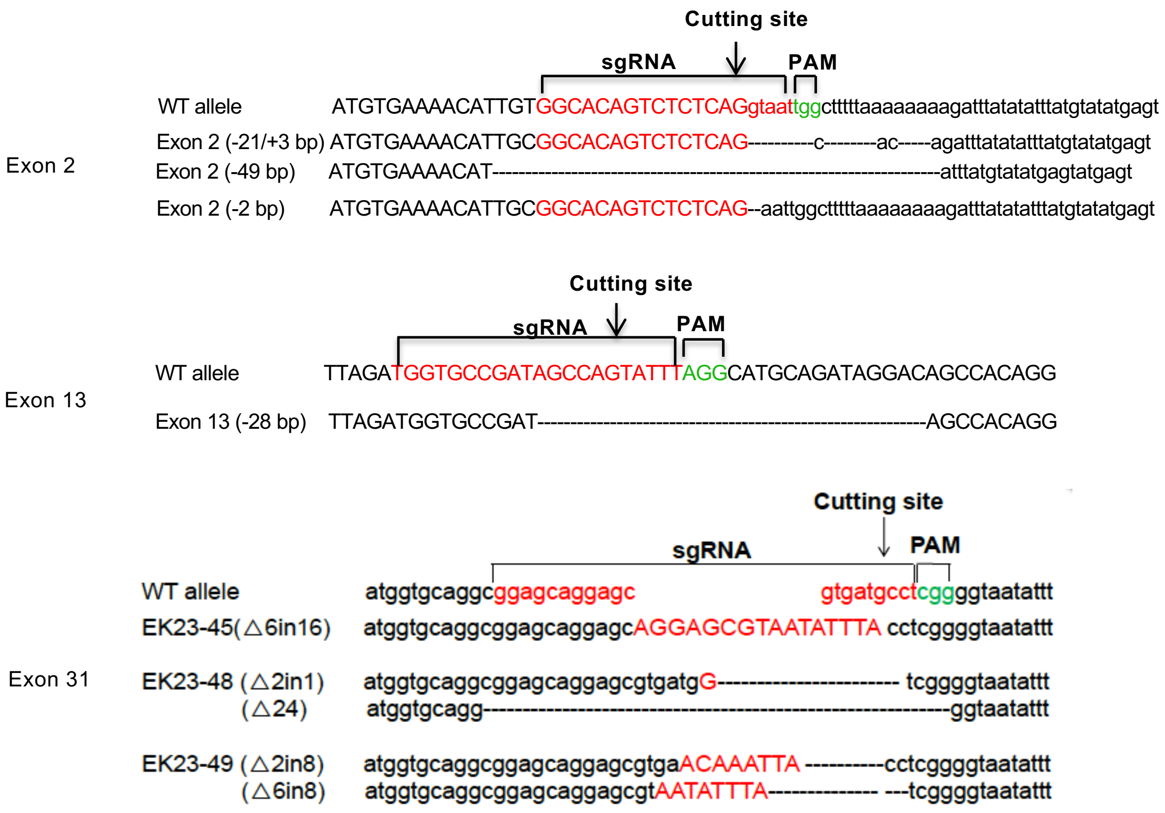

Supplementary Figure 1. Truncation of endogenous mutant *HTT* gene in HD KI mice via CRISPR/Cas9. Sequencing verification of mutations in exon 2, exon 13, and exon 31 of the mouse *HTT* gene. These mutations result in N-terminal HTT or truncated HTT fragments. gRNA sequences and indel mutations are indicated.

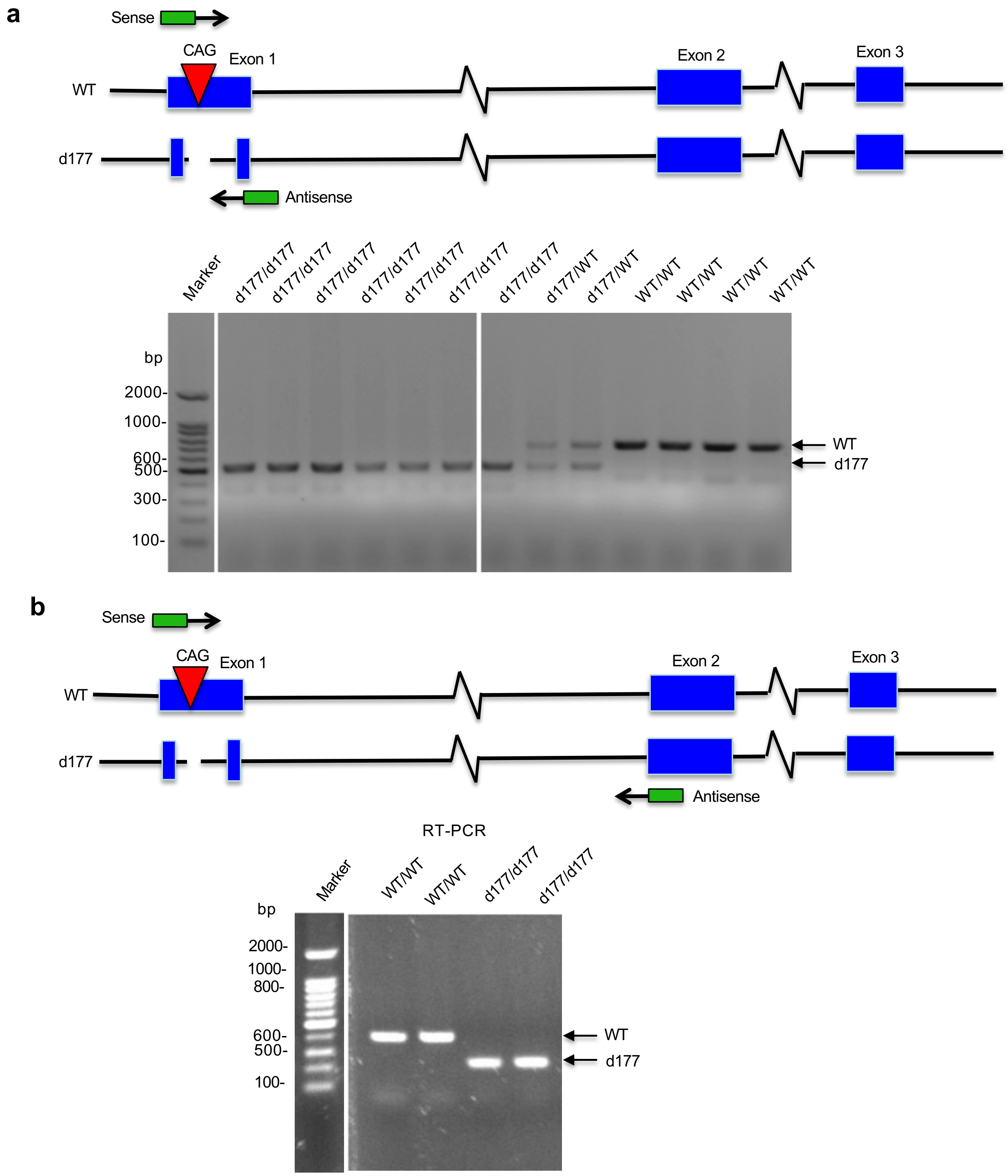

Supplementary Figure 2. d177 mutation with an in-frame deletion of exon 1 in the mouse *HTT* gene and the RNA expression of d177 mutant HTT. a PCR with primers for exon 1 showing the deletion of exon 1 *HTT* in d177 mutant mouse genome. **b** RT-PCR using primers specific for exon 1 and exon 2 showing that d177 mRNA is expressed at the same level as wild type *HTT* mRNA. More than three experiments were performed independently for RT-PCR analyses.

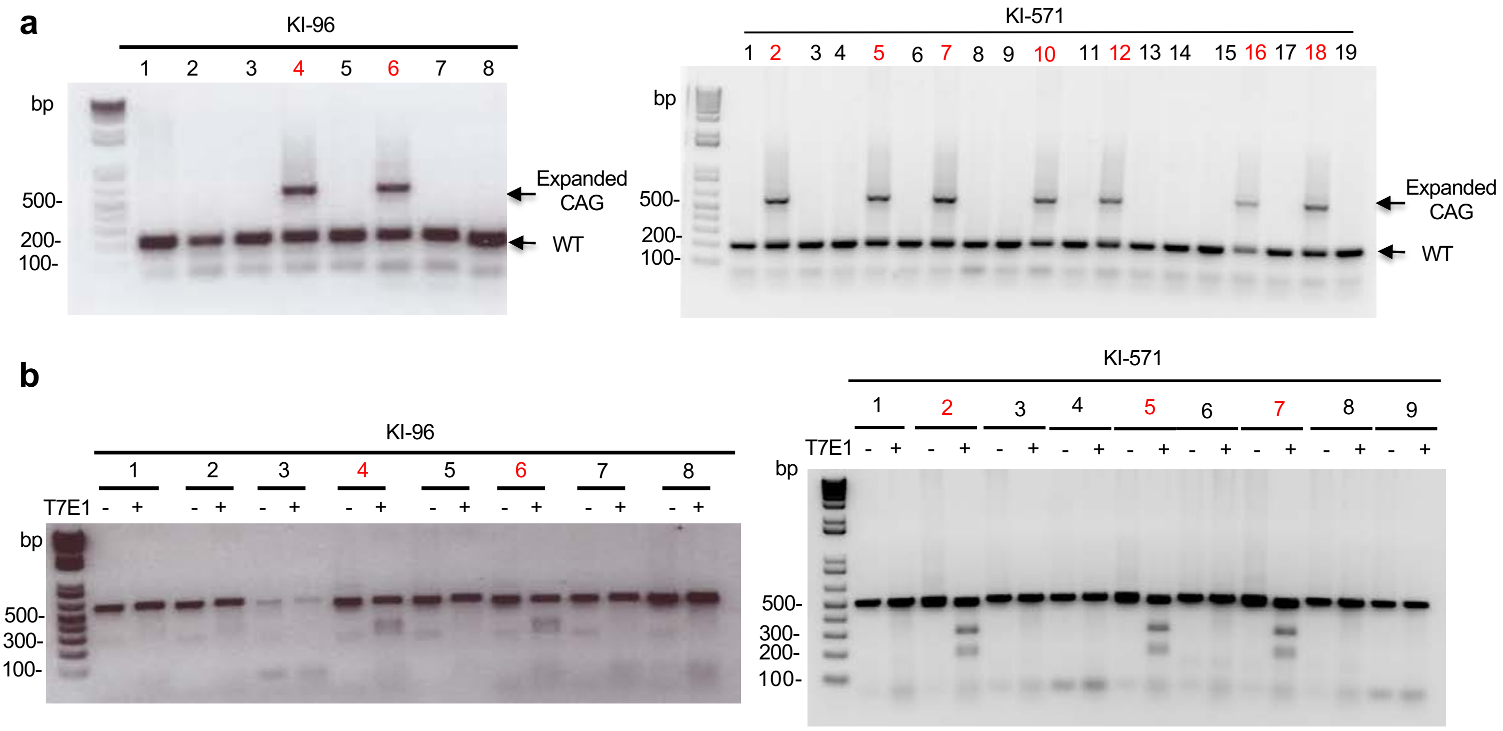

Supplementary Figure 3. T7E1 assays verified mutations in the *HTT* gene in KI-96 and KI-571 mice. a PCR revealing wild type and expanded CAG-containing *HTT* alleles in KI-96 and KI-571 mouse genomes. Red numbers indicated the presence of expanded CAG repeats. **b** Examples of T7E1 assay showing digestion of the mutant *HTT* gene in KI-96 and KI-571 mice. The numbers in red indicate the DNA samples containing mismatched expanded CAG such that the DNAs were digested. In **a** and **b**, more than three independent experiments were performed.

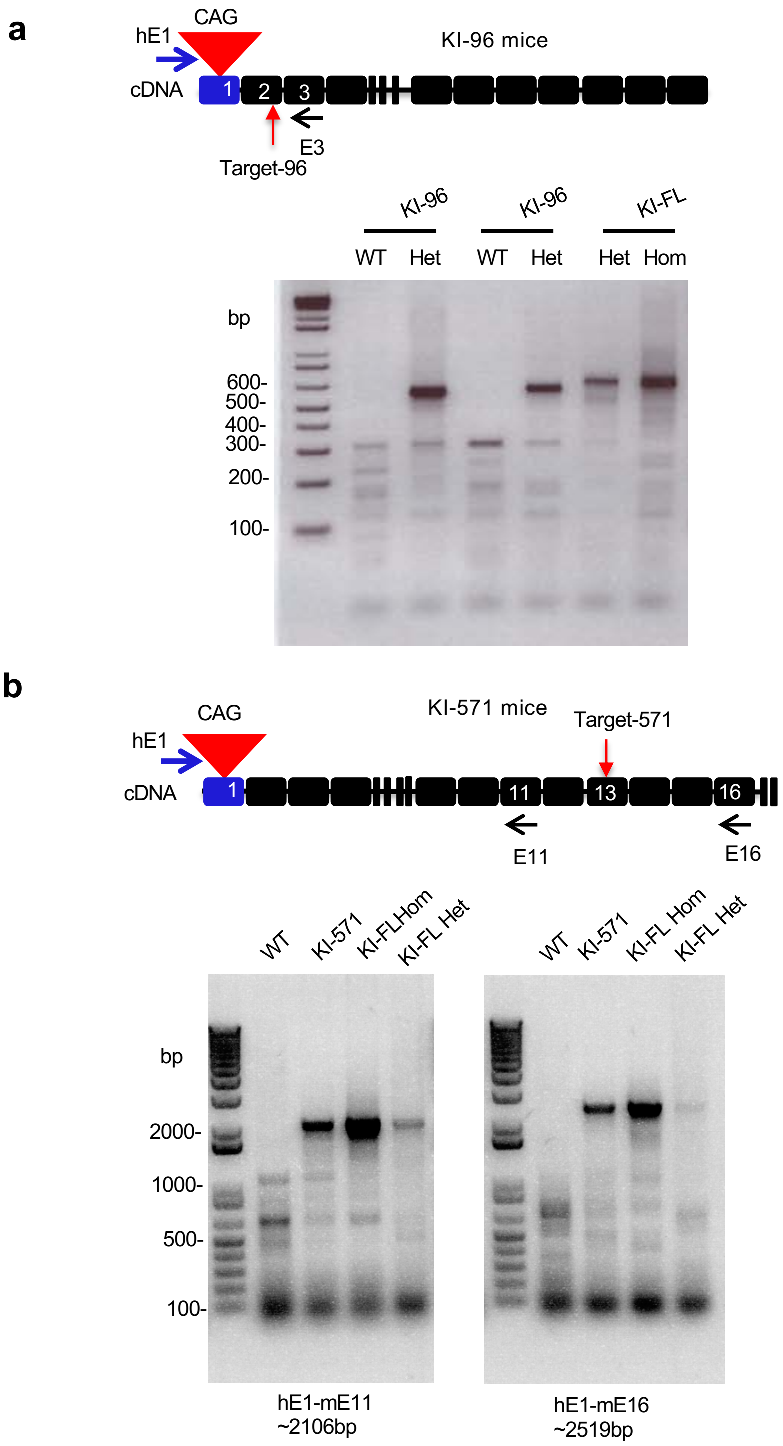

Supplementary Figure 4. Expression of mutant *HTT* RNA in KI-96 and KI-571 mouse brains. a RT-PCR showing the expression of mutant *HTT* in heterozygous KI-96 mouse striatum. The control is wild type and heterozygous or homozygous KI-FL mouse striatal tissues. Primers for exon 1 and exon 3 were used for PCR and can avoid PCR products from genomic DNAs. **b** RT-PCR showing the expression of mutant *HTT* in heterozygous KI-571 mouse striatum. The control is wild type and heterozygous or homozygous KI-FL (full-length HTT) mouse striatal tissues. Primers for exon 11 and exon 16 were used to avoid PCR products from genomic DNAs. Note that wild type mouse *HTT* cannot be amplified with human *HTT* specific primer hE1. Het, heterozygous; Hom, homozygous. The data were obtained from more than three independent experiments.

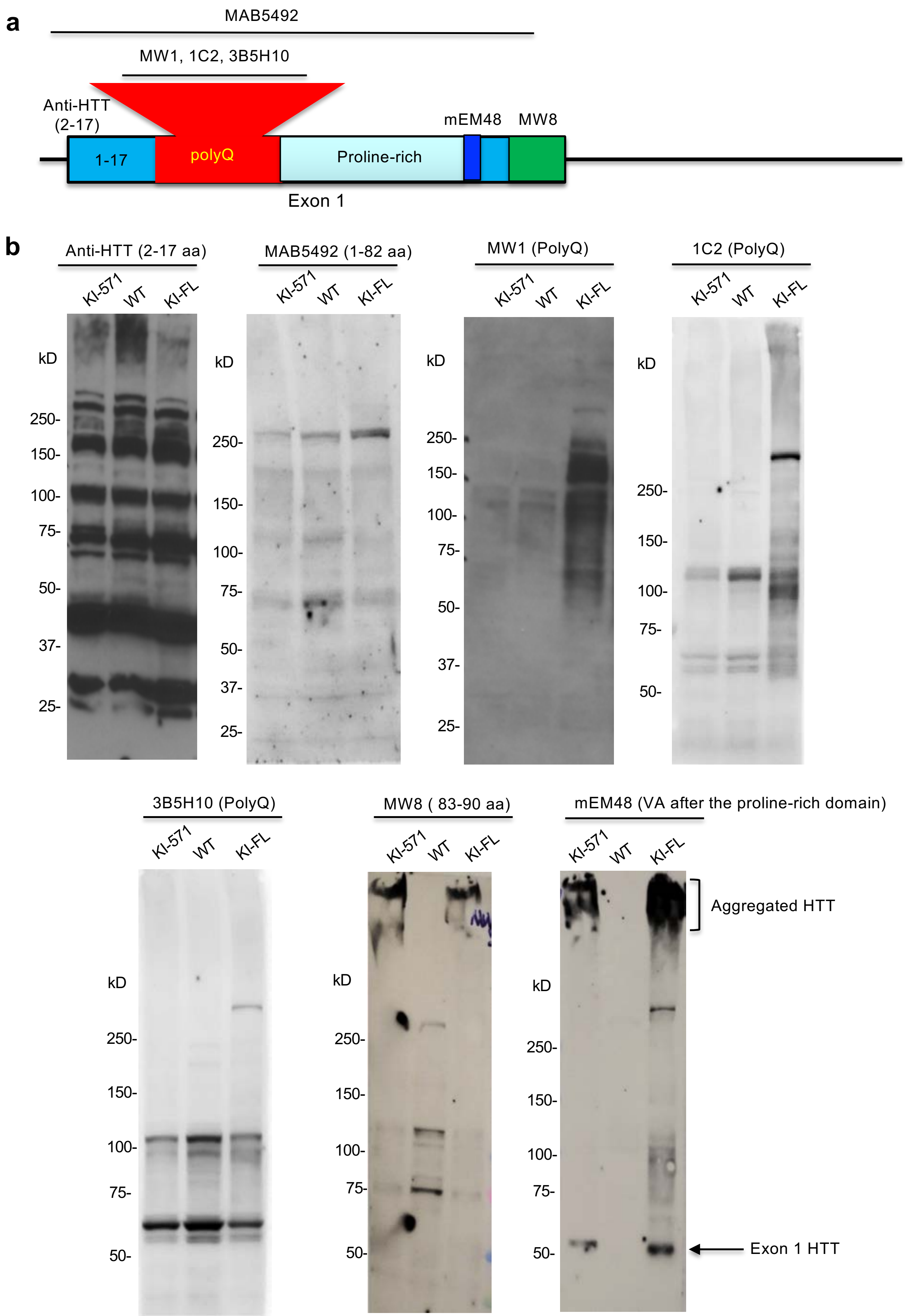

Supplementary Figure 5. Western blotting analysis of KI mouse striatal tissues using various antibodies. **a** Antibodies used in the study and their epitopes in the mouse HTT protein. **b** Western blotting with the antibodies indicated in the figure showing that only mEM48 was able to selectively recognize the stable exon 1 HTT (arrow) in KI-571 mouse striatum. Controls include wild type and HD140Q KI (KI-FL) mouse striatal tissues.

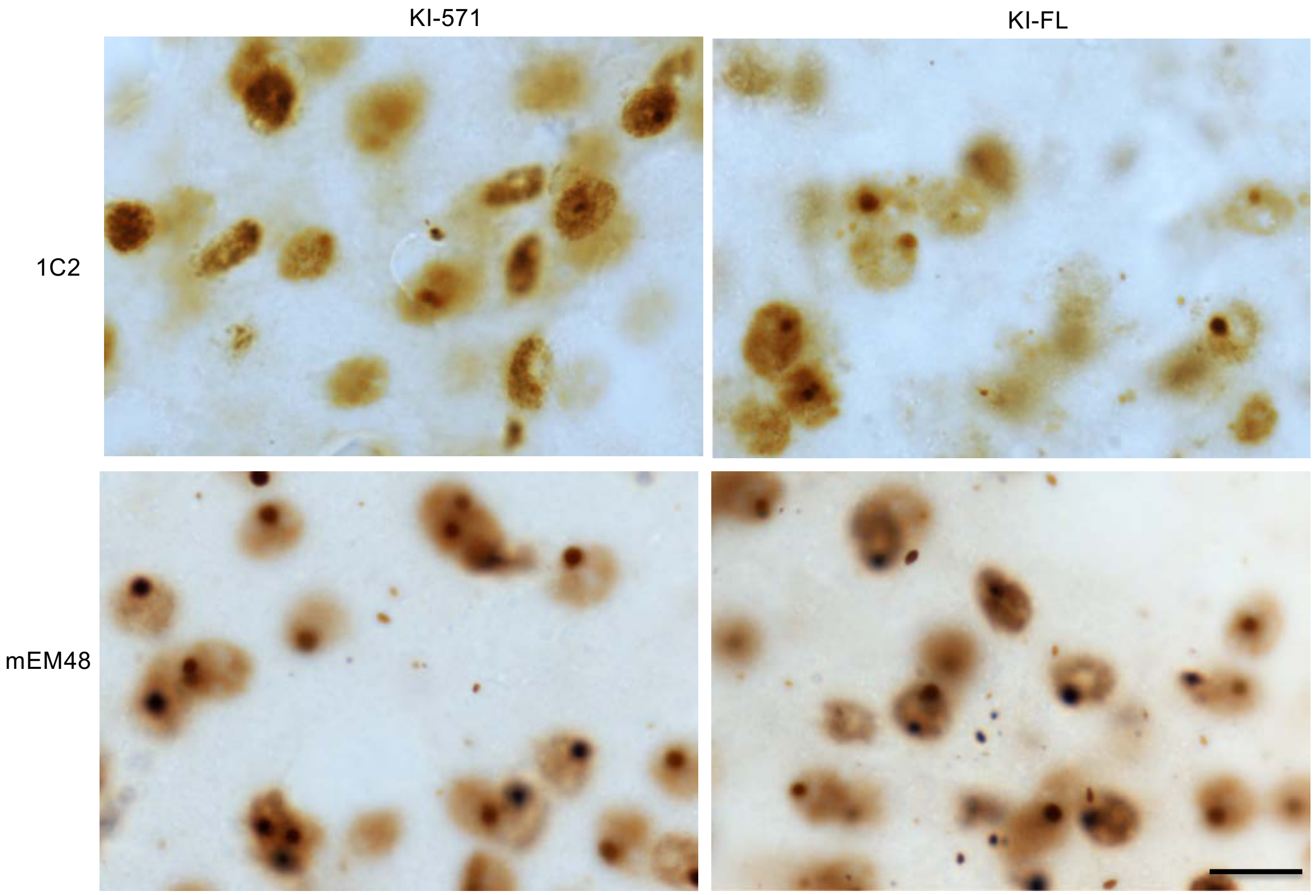

Supplementary Figure 6. Immunocytochemical staining of the striatum of KI mouse brains. The striatum of HD140Q KI (KI-FL) and KI-571 mice at 11 months of age was stained with antibodies mEM48 and 1C2. Scale bar: 15 μm.

a

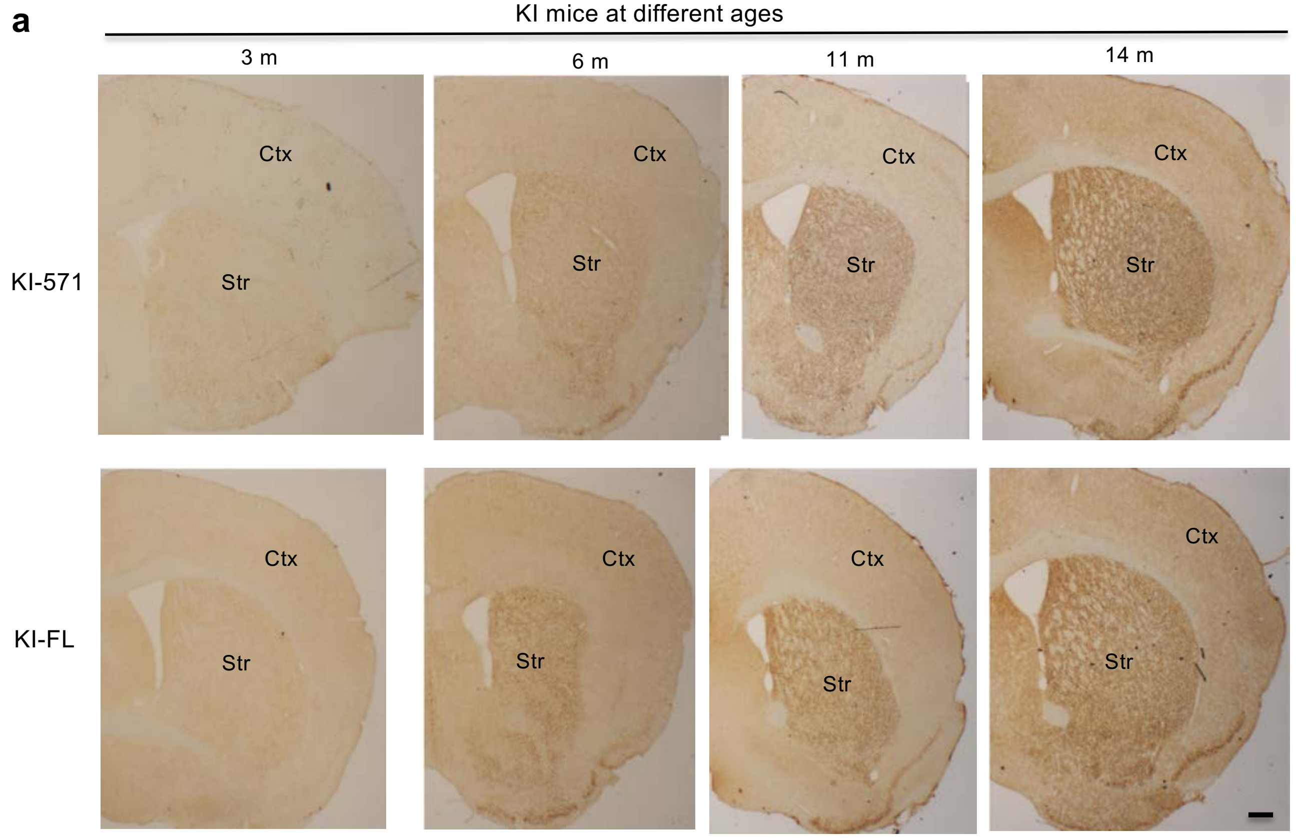

b

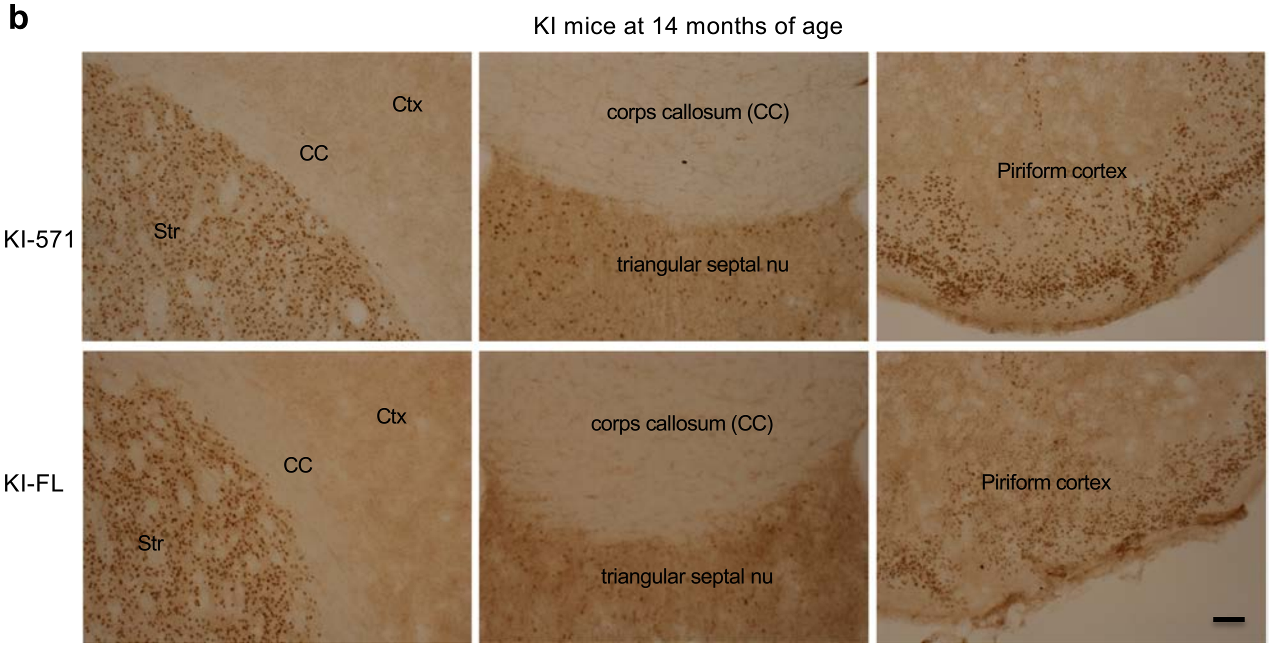

Supplementary Figure 7. Age-dependent and selective accumulation of mutant HTT in the striatum in KI-571 and KI-FL mice. a Comparison of mutant HTT distribution in different brain regions in KI-571 and KI-FL mice at different ages. Scale bar: 200 μm. **b** Similar extent and distribution of nuclear accumulation of mutant HTT in KI-571 and KI-FL mice at 14 months of age. Scale bar: 100 μm.

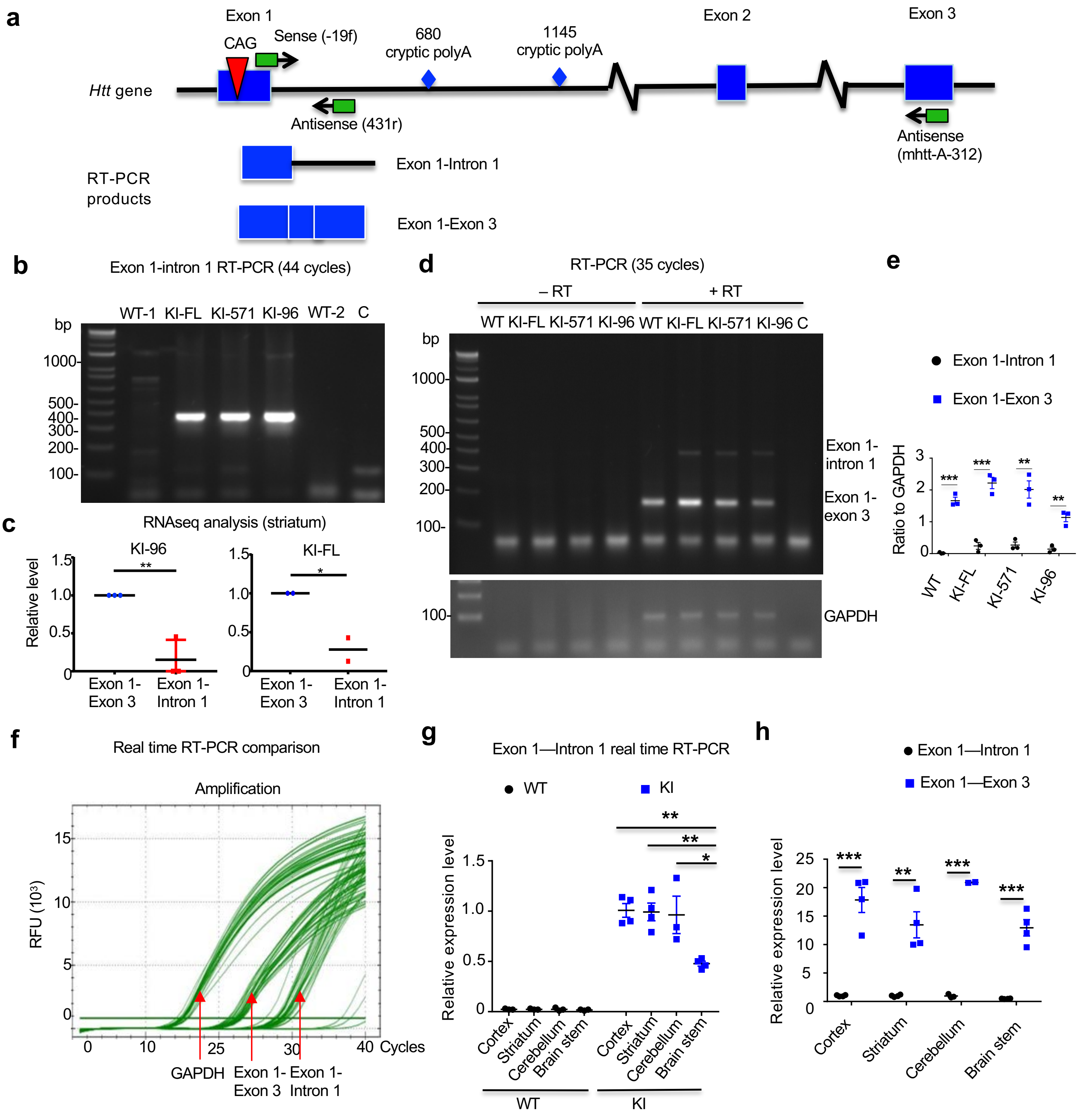
a
Exon 1
CAG
Sense (-19f)
680 cryptic polyA
1145 cryptic polyA
Exon 2
Exon 3
Htt gene
Antisense (431r)
Antisense (mhtt-A-312)
RT-PCR products
Exon 1-Intron 1
Exon 1-Exon 3
b
Exon 1-intron 1 RT-PCR (44 cycles)
bp
WT-1 KI-FL KI-571 KI-96 WT-2 C
1000-
500-
400-
300-
200-
100-
c
RNAseq analysis (striatum)
KI-96
KI-FL
Relative level
Exon 1-Exon 3
Exon 1-Intron 1
d
RT-PCR (35 cycles)
– RT
+ RT
WT KI-FL KI-571 KI-96 WT KI-FL KI-571 KI-96 C
Exon 1-intron 1
Exon 1-exon 3
GAPDH
e
Exon 1-Intron 1
Exon 1-Exon 3
Ratio to GAPDH
WT
KI-FL
KI-571
KI-96
f
Real time RT-PCR comparison
Amplification
RFU (10³)
Cycles
GAPDH
Exon 1-Exon 3
Exon 1-Intron 1
g
Exon 1—Intron 1 real time RT-PCR
WT
KI
Relative expression level
Cortex
Striatum
Cerebellum
Brain stem
h
Exon 1—Intron 1
Exon 1—Exon 3
Relative expression level
Cortex
Striatum
Cerebellum
Brain stem

Supplementary Figure 8. Comparison of mutant *HTT* mRNA expression in different KI mouse striatum. **a** PCR primers used for RT-PCR to determine the expression of mutant *HTT*. **b** RT-PCR of the incomplete spliced *HTT* (exon 1-intron 1) in WT and KI-FL mouse striatum. **c** RNAseq analysis of read counts of exon 1-exon 3 and exon 1-intron 1 sequences in KI-96 (n = 3) and KI-FL (n = 2) mouse striatum. **d** RT-PCR results showing mutant *HTT* mRNA expression in different KI mouse striatum. Controls are RT-PCR without reverse transcriptase, which can rule out products from genomic DNA amplification. **e** Quantitative data of the relative expression of mutant *HTT* mRNA (ratio to GAPDH), which were obtained from RT-PCR in (d) (n = 3, **P < 0.01, ***P < 0.001, 2-tailed student t test, WT, t = 14.77, P < 0.001; KI-FL, t = 9.482, P < 0.001; KI-571, t = 6.077, P = 0.0037; KI-96, t = 6.72, P = 0.0025). The data are presented as mean ± SEM. **f** Real time PCR results showing the amplification of cDNAs for GAPDH, exon 1-exon 3 or exon 1-intron 1 *HTT*. **g** Comparison of WT and KI-FL mice for the expression levels of exon 1-intron 1 detected by RT-PCR (n = 3 for WT group, n = 4 for KI group, **P < 0.01, ***P < 0.001, one-way ANOVA with Turkey post-tests comparing exon1-intron1 level in different brain regions in KI, F = 7.793, P = 0.0046). The data are presented as mean ± SEM. **h** Comparison of exon 1-exon 3 and exon 1-intron 1 *HTT* levels in different brain regions in KI-FL mice. The data were obtained from 3 independent experiments of 4 KI-FL mice, **P < 0.01, ***P < 0.001, 2-tailed student t test, cortex, t = 7.658, P < 0.001; striatum, t = 5.455, P = 0.00158; cerebellum, t = 81.42, P < 0.001; brain stem, t = 8.669, P < 0.001). The data are presented as mean ± SEM.

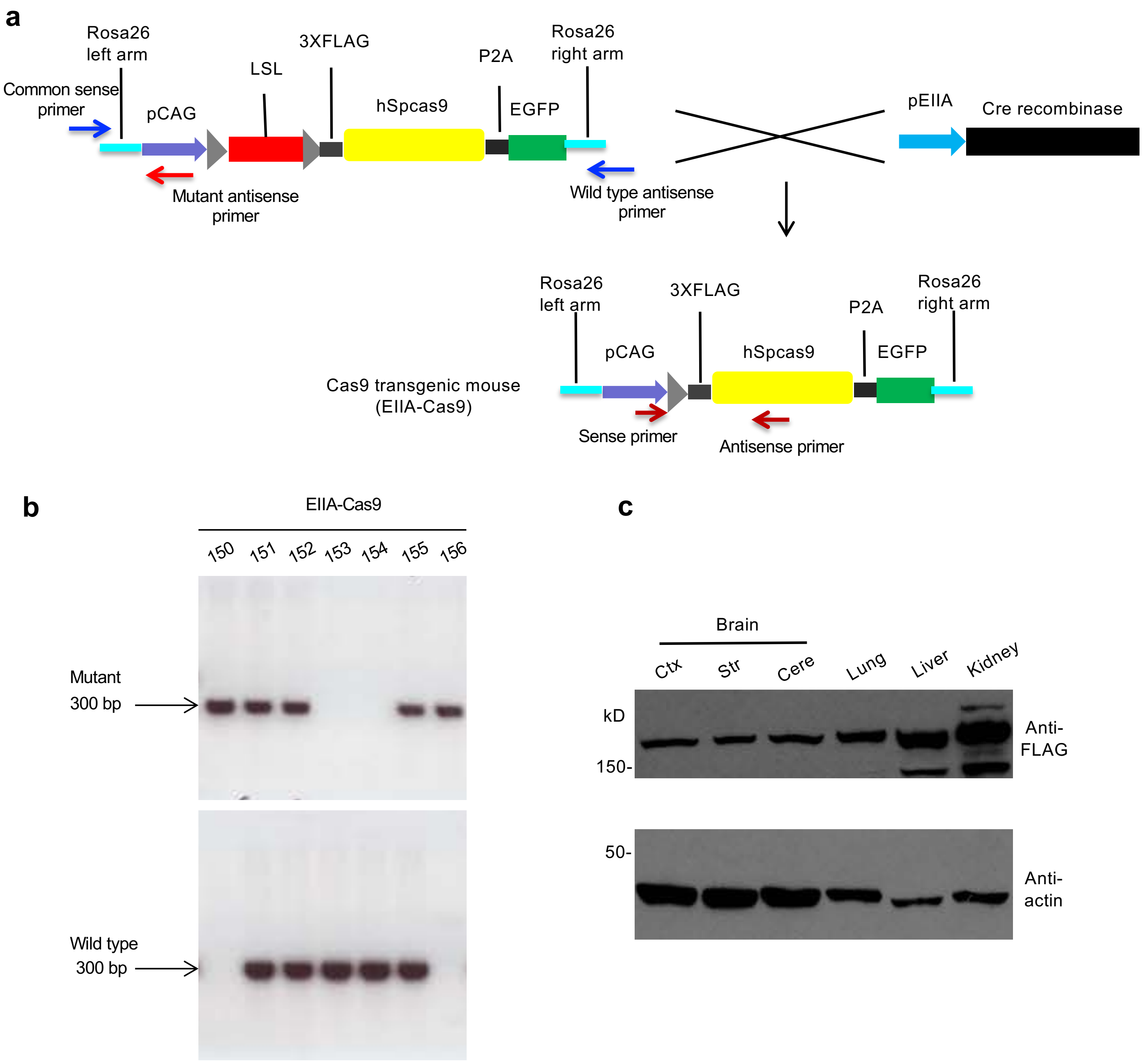

Supplementary Figure 9. Generation of HD140Q KI/Cas9 (KI/Cas9) mice. a Conditional Cas9-flag mice were crossed with EIIA-Cre transgenic mice to generate offspring in which Cre recombination has removed the inhibitory LSL to allow the expression of Cas9-flag ubiquitously under the pCAG promoter. **b** PCR genotyping verified the presence of transgenic Cas9 in KI/Cas9 mice. **c** Western blotting of KI/Cas9 mouse brain tissues showing the expression of Cas9-flag. The blot was probed with anti-flag and anti-actin.

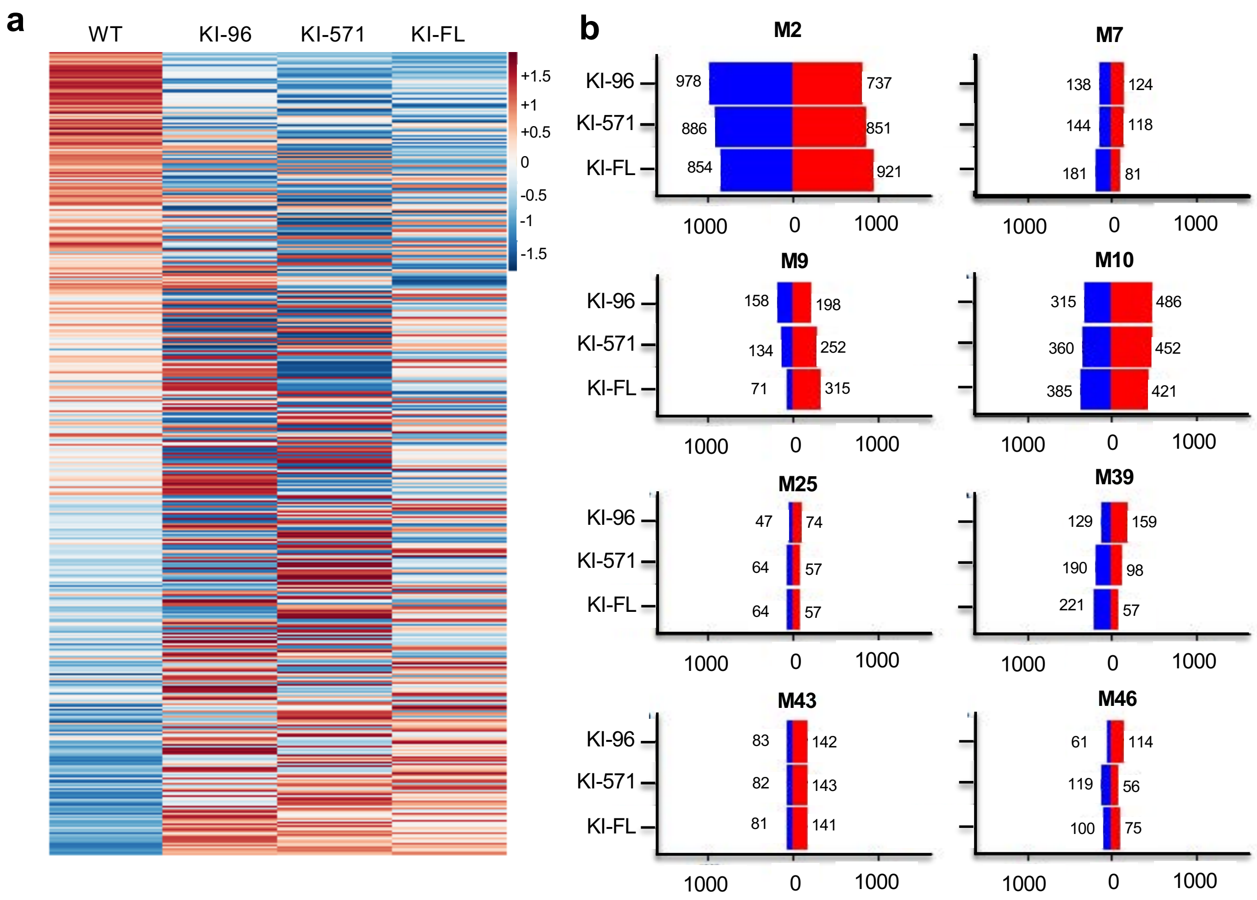

Supplementary Figure 10. Similar alterations in gene transcription in all KI mouse striatum. a Heat map representing gene expression patterns of differential expression genes when comparing WT with KI-96, KI-571, and KI-FL mice. The heatmap represents normalized mean values of a total of 4025 genes. Each column represents data of each genotype (*n* = 4 mice per genotype). **b** Similar changes of modules in KI-96, KI-571, and KI-FL when comparing with WT. Modules related to different cellular functions (M2: repeat-associated HD signaling; M7: cell death signaling; M9: mitochondria and transport; M10: UBI conjugation; M25: glutamate receptor signaling; M39: DNA repair; M43: fatty acid catabolic process; M46: glucocorticoid signaling) were selected for comparison. The data were obtained from RNAseq analysis of the striatum in WT and HD KI mice at 6 months of age (*n* = 4 mice per genotype). The bar plot was generated based on averages of log10 p-value of the grouped genes in different modules (KI-FL vs. WT, KI-96 vs. WT and KI-571 vs. WT).

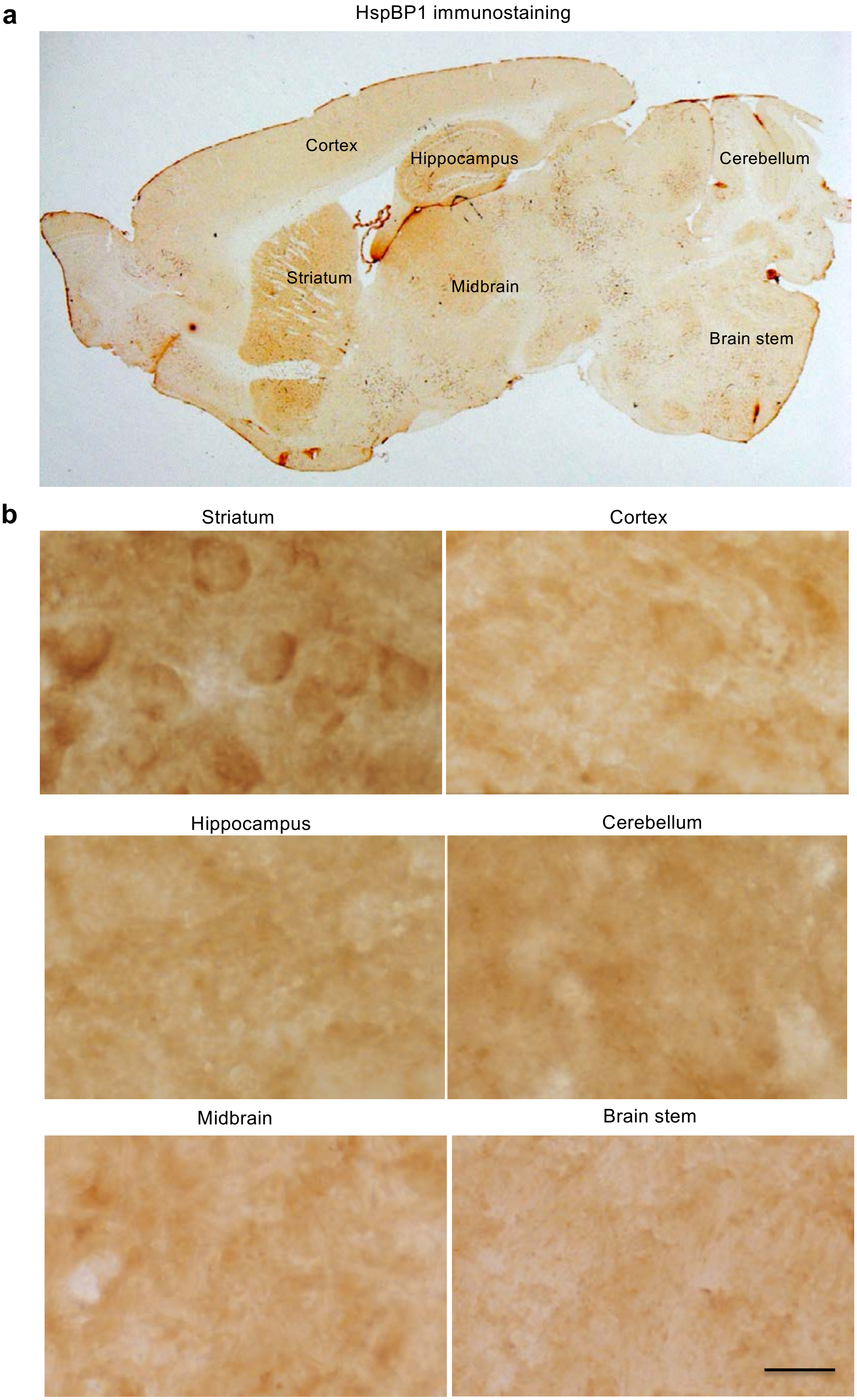

Supplementary Figure 11. HspBP1 immunostaining of the mouse brain. a, b Low (a) and high (b) magnification micrographs showing that HspBP1 is enriched in the neuronal cells in the striatum of a WT mouse at the age of 6 months. Scale bar: 15 μm.

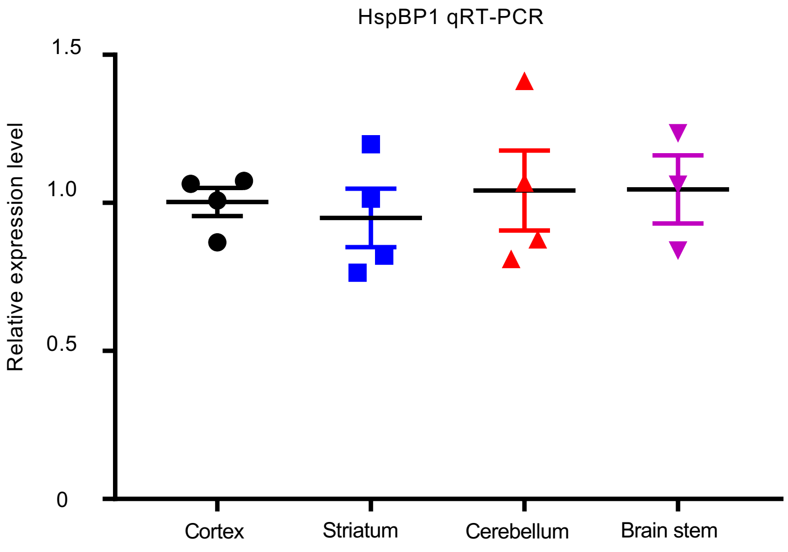

Supplementary Figure 12. HspBP1 RT-PCR showing that HspBP1 mRNA expression in different mouse brain regions is at similar levels. The data are mean ± SEM and were obtained from 3 independent experiments using 4 wild type mice at 2.5 months of age (*$P < 0.05$, ***$P < 0.001$, one-way ANOVA with Tukey post-tests, $F = 0.1866$, $P = 0.9033$).

Supplementary tables

Supplementary Table 1

REAGENT or RESOURCE	SOURCE	IDENTIFIER
**Antibodies**		
Mouse monoclonal anti-huntingtin, clone mEM48	Chemicon	Cat# MAB5374
Mouse monoclonal anti-huntingtin, clone MW8	DSHB	Cat# AB_528297
Mouse monoclonal anti-polyglutamine, clone 3B5H10	Sigma	Cat# P1874
Mouse monoclonal anti-polyglutamine, clone 5TF1-1C2	Chemicon	Cat# MAB1574
Mouse monoclonal anti-huntingtin, clone HU_2E8	Chemicon	Cat# MAB2166
Mouse monoclonal anti-huntingtin	Chemicon	Cat# MAB5492
Anti-HTT (2-17)	(Enzo Life)	Cat# BML-PW0595
Mouse monoclonal anti-GAPDH	Chemicon	Cat# MAB374
Mouse monoclonal anti-Vinculin	Sigma	Cat# V9131
Rabbit polyclonal anti-Actin	Sigma	Cat# A5060
Mouse monoclonal anti-γ tubulin	Sigma	Cat# T6557
Rabbit polyclonal anti-GFAP	Thermo Fisher	Cat# RB-087-A
Mouse monoclonal anti-FLAG	Sigma	Cat# F1804
Rabbit polyclonal anti-GFP	Sigma	Cat# G1544
Rabbit polyclonal anti-RFP	MBL	Cat# PM005
Donkey Anti-Rabbit	Jackson Immunolabs	Cat# 715-035-152
Donkey Anti-Mouse	Jackson Immunolabs	Cat# 715-035-151
Goat Anti-Rabbit IgG (H+L) Biotinylated	Vector Labs	Cat# BA-1000
Goat Anti-Mouse IgG (H+L) Biotinylated	Vector Labs	Cat# BA-9200
DAPI	Sigma	Cat# D9542
Goat anti-Rabbit IgG (H+L) Secondary Antibody, Alexa Fluor 488	Thermo Fisher	Cat # A-11034
Mouse anti-HspBP1<br>Rabbit anti-HspBP1	Santa Cruz Biotech<br>Sigma	Cat# 390467 (for WB)<br>Cat#A0771444 (for ICC)

Supplementary Table 2

**Bacterial and Virus Strains**		
Xl1-blue	Stratagene	Cat#200249
AAV-2/9	Viral Vector Core at Emory University	N/A
**Chemicals, Peptides, and Recombinant Proteins**		
T7 Endonuclease I	NEB	Cat# M0302S
**Critical Commercial Assays**		
ECL™ Prime Western Blotting System	GE Healthcare	Cat# RPN222
VECTASTAIN Elite ABC Kits	Vector	Cat# PK-2200

Supplementary Table 3

gRNA sequence and targeting exons

Name of sgRNA	mRNA locus	Amino acid locus	Exon locus	Sequence (PAM sequence are shown in red)
Human HTT-T1			E1	GGCCTTCATCAGCTTTTCCAGGG
Mouse HTT-96	272-287	91-96(AA)	E2	GGCACAGTCTCTCAGGTAATTGG
Mouse HTT-571	1692-1715	564-572(AA)	E13	GGTGCCGATAGCCAGTATTTAGG
Mouse HTT-(d177)		56 aa deletion	E1-T1<br>E1-T3	CCCTGGAAAAGCTGATGAAGGC<br>CCAGGTCCGGCAGAGGAACCGC
Mouse HTT-1367		1295-1369 (AA)	E31	GGAGCAGGAGCGTGATGCCTCGG
Mouse HspBP1			E2	GGGGGCAGTGGTTCCTCGGCGGGGGG
Control gRNA				ACC GGA AGA GCG ACC TCT TCT

Supplementary Table 4

Oligonucleotides

Name of primer	Sequence	Function
msHTT-s-94	GCTGCTAAGTGGCGCCGCGTAG	PCR genotyping, RT-PCR
HD01#3	GCGGCTGAGGGGGTTGA	PCR genotyping, RT-PCR
hHDA177	GAGGCAGCAGCGGCTGTGCCTG	PCR genotyping, RT-PCR / qRT-PCR
HD4(25)	ATGGCCTTCGAGTCCCTCAAGT	PCR genotyping, RT-PCR / qRT-PCR
HTT-571 F1	TAATACGACTCACTATAGGTGCCGATAGCCAGTA	In vitro sgRNA mRNA synthesis
HTT-571 R1	TTCTAGCTCTAAAACAAATACTGGCTATCGGCAC	In vitro sgRNA mRNA synthesis
HTT-96 F1	TAATACGACTCACTATAGGCACAGTCTCTCAGGT	In vitro sgRNA mRNA synthesis
HTT-96 R1	TTCTAGCTCTAAAACATTACCTGAGAGACTGTGC	In vitro sgRNA mRNA synthesis
T1-F1	TAATACGACTCACTATAG GCCTTCATCAGCTTTTCCA	In vitro sgRNA mRNA synthesis
T1-R1	TTCTAGCTCTAAAACTGGAAAAGCTGATGAAGGC	In vitro sgRNA mRNA synthesis
Mouse sgRNA-571S	ACCGGTGCCGATAGCCAGTATTT	DNA recombination for AAV plasmid
Mouse sgRNA-571A	AACAAATACTGGCTATCGGCACC	DNA recombination for AAV plasmid
Mouse sgRNA-91S	ACCAACAATATGTGAAAACATTG	DNA recombination for AAV plasmid
Mouse sgRNA-91A	AACCAATGTTTTCACATATTGTT	DNA recombination for AAV plasmid
U6-For	GAGGGCCTATTTCCCATGATTC	Primer for sequencing
HTT-571 For	TGCATTTACCTGTGGCCACTG	PCR for T7E1assay
HTT-571 Rev	TCGCTGGCTAGTGGTGGTTC	PCR for T7E1assay
HTT-96-For	ATCTGTCGTCATCCCTTCCT	PCR for T7E1assay
HTT-96-Rev	TGGTACTGGCTCTGTAGTC	PCR for T7E1assay
mHTT-E11-R	TGTCCGAAGGAGTCACAGCTGA	RT-PCR
mHTT-E13-R	TCCTGTGGCTATCGGCACCA	RT-PCR
mHTT-E16-R	AGGGCCTTCACACTGACTCTC	RT-PCR
-19f	AGGAACCGCTGCACCGA	RT-PCR
431r	GAGACCTCCTAAAAGCATTATGTCATC	RT-PCR
hHD-E1-S	TGGCTGAGGAGCCGCTGCA	RT-PCR
mHD-E3-A	TCCATAGCGATGCCCAAGAG	RT-PCR

Lop Cas9 For	ACGTGCTGGTTATTGTGCTGTC	PCR genotyping
Lop Cas9 Rev	TTCTTCTGGCGGTTCTCTTCAG	PCR genotyping
Cas9 Common Sense	AAGGGAGCTGCAGTGGAGTA	PCR genotyping
Cas9 Mut reverse	CGGGCCATTTACCGTAAG TTAT	PCR genotyping
Cas9 WT reverse	CCGAAAATCTGTGGGAAGTC	PCR genotyping
GAPDH-141S	ACGACCCCTTCATTGACCTC	RT-PCR
GAPDH-498A	GGGGGCTAAGCAGTTGGTGG	RT-PCR
GAPDH-S	TGTTTCCTCGTCCCGTAGAC	qRT-PCR
GAPDH-A	AATCTCCACTTTGCCACTGC	qRT-PCR
HspBP1-S2	ATCTGCTGGTGGGTCGATAC	qRT-PCR
HspBP1-A2	CAACACCTGCTCCTGGATG	qRT-PCR
d177 forward	ACTGCTAAGTGGCGCCGCGTAG	PCR genotyping
d177 reverse	AGGAGGTAACCCTAGAGATCTCTGC	PCR genotyping
Exon31 forward	CTAACCGCTGTCCACTAGCTGGC	PCR genotyping
Exon31 reverse	CTGTCACCTCTGGATAGCTGACTAC	PCR genotyping
