# Supplementary material for: Truncation of mutant huntingtin in knock-in mice demonstrates exon1 huntingtin is a key pathogenic form
Source: Nat Commun. 2020 May 22;11:2582. doi: 10.1038/s41467-020-16318-1 (PMC7244548; doi:10.1038/s41467-020-16318-1)
Supplement: Supplementary file 3 — Reporting summary [file 41467_2020_16318_MOESM3_ESM.pdf]

nature research

Corresponding author(s): Xiao-Jiang Li

Last updated by author(s): Apr 12, 2020

Reporting Summary

Nature Research wishes to improve the reproducibility of the work that we publish. This form provides structure for consistency and transparency in reporting. For further information on Nature Research policies, seeAuthors & Referees and theEditorial Policy Checklist .

Statistics

For all statistical analyses, confirm that the following items are present in the figure legend, table legend, main text, or Methods section.

n/a	Confirmed	
☐	☒	The exact sample size (*n*) for each experimental group/condition, given as a discrete number and unit of measurement
☐	☒	A statement on whether measurements were taken from distinct samples or whether the same sample was measured repeatedly
☐	☒	The statistical test(s) used AND whether they are one- or two-sided<br>*Only common tests should be described solely by name; describe more complex techniques in the Methods section.*
☒	☐	A description of all covariates tested
☐	☒	A description of any assumptions or corrections, such as tests of normality and adjustment for multiple comparisons
☒	☐	A full description of the statistical parameters including central tendency (e.g. means) or other basic estimates (e.g. regression coefficient) AND variation (e.g. standard deviation) or associated estimates of uncertainty (e.g. confidence intervals)
☐	☒	For null hypothesis testing, the test statistic (e.g. *F*, *t*, *r*) with confidence intervals, effect sizes, degrees of freedom and *P* value noted<br>*Give P values as exact values whenever suitable.*
☒	☐	For Bayesian analysis, information on the choice of priors and Markov chain Monte Carlo settings
☒	☐	For hierarchical and complex designs, identification of the appropriate level for tests and full reporting of outcomes
☒	☐	Estimates of effect sizes (e.g. Cohen's *d*, Pearson's *r*), indicating how they were calculated

Our web collection on statistics for biologists contains articles on many of the points above.

Software and code

Policy information about availability of computer code

Data collection	Softwares used in RNA sequencing were described in methods. Guide RNA was designed by an online tool (https://www.benchling.com/crispr/)
Data analysis	statistical analyses were performed using the Prism 7 (GraphPad) software.

For manuscripts utilizing custom algorithms or software that are central to the research but not yet described in published literature, software must be made available to editors/reviewers. We strongly encourage code deposition in a community repository (e.g. GitHub). See the Nature Research guidelines for submitting code & software for further information.

Data

Policy information about availability of data

All manuscripts must include a data availability statement. This statement should provide the following information, where applicable:

- Accession codes, unique identifiers, or web links for publicly available datasets
- A list of figures that have associated raw data
- A description of any restrictions on data availability

The data generated or analyzed during the current study are available within the article, supplementary information, attached source data file, and from the corresponding author upon reasonable request. The RNA sequencing data have been deposited in the Gene Expression Omnibus (GEO) database and the accession code is GSE145879. The data that support the findings of this study are available from the corresponding author upon reasonable request.

Field-specific reporting

Please select the one below that is the best fit for your research. If you are not sure, read the appropriate sections before making your selection.

Life sciences study design

All studies must disclose on these points even when the disclosure is negative.

Sample size	For biochemical and histological studies, we used at least 3 mice per group. For animal behavioral studies, we used at least 8 mice per genotype.
Data exclusions	No data were excluded from the analyses.
Replication	For immunostaining, western blotting, RT-PCR and qPCR, samples from at least three mice were used, and the results were analyzed from at least three independent experiments. Additional animals were used or additional experiments were performed if the prior experiments were not done under the same conditions.
Randomization	Animals used for each experiment are littermates randomly assigned to the experimental groups.
Blinding	RNA sequencing analyses were performed by personnel blinded to the treatment conditions. For western blotting, immunostaining, RT-PCR/ qPCR, no blinding was used because the samples needed to be treated with different reagents.

Reporting for specific materials, systems and methods

We require information from authors about some types of materials, experimental systems and methods used in many studies. Here, indicate whether each material, system or method listed is relevant to your study. If you are not sure if a list item applies to your research, read the appropriate section before selecting a response.

Materials & experimental systems

n/a	Involved in the study	
☐	☒	Antibodies
☒	☐	Eukaryotic cell lines
☒	☐	Palaeontology
☐	☒	Animals and other organisms
☒	☐	Human research participants
☒	☐	Clinical data

Methods

n/a	Involved in the study	
☒	☐	ChIP-seq
☒	☐	Flow cytometry
☒	☐	MRI-based neuroimaging

Antibodies

Antibodies used	Primary antibodies used in this study include: mEM48(Chemicon, MAB5374), MW8(DSHB, AB_528297), 3B5H10(Sigma, P1874), 1C2(Chemicon, MAB1574), 2166(Chemicon, MAB2166), MAB5492(Chemicon, MAB5492), Anti-HTT (2-17) (Enzo life, BML-PW0595), GAPDH(Chemicon, MAB374), Vinculin(Sigma, V9131), Actin(Sigma, A5060), γ tubulin(Sigma, T6557), GFAP(Thermo Fisher, RB-087-A), FLAG(Sigma, F1804), GFP(Sigma, G1544), RFP(MBL, PM005). Secondary antibodies were donkey anti-rabbit, donkey anti-mouse Alexa Fluor 488 or 594 from Jackson ImmunoResearch.
Validation	All antibodies used in this study have been validated by manufacturers or validated in published scientific literature.

Animals and other organisms

Policy information about studies involving animals; ARRIVE guidelines recommended for reporting animal research

Laboratory animals	The generation of new KI mice was described in details in the method section. Wild type C57BL/6 mice, HD 140Q germline HD knock-in mice have been stably maintained in our lab. Cas9 transgenic mice were generated by crossing EIIa-Cre mice with conditional Cas9 mice that were obtained from the Jackson Laboratory . Both male and female mice were involved in this study. The ages of mice are from 3 to 11 months and are indicated in the text or figure legends.
Wild animals	This study did not involve wild animals
Field-collected samples	All mice were maintained on a 12:12 h light/dark cycle (lights off at 7 p.m.). The temperature was maintained at 22 ± 1 °C with relative humidity (30-70%).
Ethics oversight	All animal procedures were approved by the Institutional Animal Care and Use Committee of Emory University.

Note that full information on the approval of the study protocol must also be provided in the manuscript.
